# Internal and external load during on-field training drills with an aim of improving the physical performance of players in professional soccer: a retrospective observational study

**DOI:** 10.3389/fphys.2023.1212573

**Published:** 2023-11-07

**Authors:** Linda Ammann, Paweł Chmura

**Affiliations:** ^1^ Independent Researcher, Luzern, Switzerland; ^2^ Department of Team Games, Wroclaw University of Health and Sport Sciences, Wrocław, Poland

**Keywords:** load management, soccer, training, drills, sided games, HIIT, physical performance, team sports

## Abstract

Extensive research has led to evidence-based methodological recommendations for appropriate prescription and implementation of different training drills to improve the physical performance of professional soccer players, while also pointing out limitations of drills. Less is known about the current methods used in an ecological context and the extent to which evidence-based considerations are applied. Knowledge of current practices might also enable to identify pitfalls in successful implementation and/or deficiencies in the communication of scientific knowledge. Thus, the aim of this study was to quantify and compare the load that players experience in an ecological context during drills that are frequently used, and in which there is an intention to improve the players’ physical capacities. Therefore, a retrospective observational cohort study was conducted over a 14-month period, analyzing 9 load measures during training drills of 39 players of a team competing in the highest Swiss league. The load experienced by players was statistically significant different between the assessed drill categories for each load measure (all *p* < .001). This indicates different drills provide different stimuli. HIIT drills proved to be a more powerful tool of getting players to cover distances at high-speed and to spend time at an intensity ≥90% HR_max_ compared to sided games. The sprint distance of players was very low in all sided games and in most cases also in HIIT drills, in the latter the players also hardly performed any deceleration. In small goal-oriented sided games, players covered a greater distance per minute when outside floaters were present. Particularly regarding an improvement of the players’ aerobic capacity, the present data emphasize the relevance for coaches to ensure an appropriate exposure. In general, the importance of individual load management in professional soccer is highlighted.

## Introduction

The performance of soccer players depends on different components, of which physical, technical, and tactical performance, as well as psychological factors are the most important ones (Julian et al., 2021; Dambroz et al., 2022; Forcher et al., 2022; Sarmento et al., 2022). Physical performance capacity, being itself one of these important constructs, is furthermore assumed to directly influence tactical, technical, and psychological components of match performance (Julian et al., 2021; Dambroz et al., 2022; Forcher et al., 2022; Sarmento et al., 2022). Therefore, soccer players should aim to optimize their physical capabilities in both the short and long term. To optimally develop the physical capabilities of soccer players, training stimuli need to be applied individually even within a team environment (Halson, 2014; Bourdon et al., 2017; Teixeira et al., 2021; Lima-Alves et al., 2022; Ammann and Altmann, 2023; Ammann et al., 2023b).

Training and competition load can be defined as an input variable that is usually tried to be manipulated when intending to elicit certain training induced adaptations (as this term including competitions) (Impellizzeri et al., 2020). Measures of load can be categorized as either external or internal, depending on whether they refer to measurable aspects occurring externally or internally to the athlete (Bourdon et al., 2017; Impellizzeri et al., 2019; Miguel et al., 2021). External loads are objective measures of the work performed by an athlete. In contrast, internal loads refer to the relative biological (both physiological and psychological) stressors imposed on the athlete (Bourdon et al., 2017; Impellizzeri et al., 2019). To individually tailor training programs, maximizing positive physiological adaptation and at the same time preventing injury and illness, careful load monitoring is required (Halson, 2014; Akenhead and Nassis, 2016; Bourdon et al., 2017; Impellizzeri et al., 2019; Miguel et al., 2021). Within load monitoring, employing an integrated load monitoring approach (i.e., rigorous, and consistent, combining both external and internal loads) seems crucial (Halson, 2014; Bourdon et al., 2017; Impellizzeri et al., 2019; Miguel et al., 2021; Ammann et al., 2023b).

So-called sided games are widely implemented training drills (here: uninterrupted training sequence in which the same method is practiced) by coaches in soccer (Hill-Haas et al., 2011; Clemente et al., 2021; Clemente et al., 2022; Dello Iacono et al., 2023). Sided games are modified games of shorter duration and played on reduced pitch areas compared to traditional soccer matches. Compared to more traditional training methods, they are characterized by (more pronounced) multi-dimensional demands. By deliberately manipulating different aspects (e.g., duration, number of games, rest intervals, pitch configuration, scoring method, rules, format), coaches can focus on the technical, tactical, physical, or psychological development of players depending on their priorities (Hill-Haas et al., 2011; Clemente et al., 2021; Clemente et al., 2022; Dello Iacono et al., 2023). Sided games have been studied extensively in recent years, resulting in, among other things, evidence-based methodological recommendations for appropriate prescription and implementation (Clemente et al., 2021; Dello Iacono et al., 2023). However, literature also points to some facts worth taking into account with regard to the holistic training process when considering employing sided games (Hill-Haas et al., 2011; Clemente et al., 2021; Clemente et al., 2022; Dello Iacono et al., 2023). Regarding the physical development of players, for example, distances covered with higher running speeds have been found to be rather low and they showed poor reliability (Clemente et al., 2022; Dello Iacono et al., 2023). Furthermore, some acute effects and adaptations are known to depend on factors such as fitness level, sex, age, expertise, technical/tactical skill level, or psychological aspects (Hill-Haas et al., 2011; Clemente et al., 2021; Clemente et al., 2022; Dello Iacono et al., 2023). Another fact, being simultaneously a strength and a (potential) weakness, is that many findings were obtained in highly controlled experimental conditions or derived from a rather small number of observations in real world professional soccer settings (also referred as ecological context), the latter being inherently limited in their generalizability (Clemente et al., 2021; Beato et al., 2023; Dello Iacono et al., 2023).

Given the aims of preparing players for the sport-specific demands (in the end the match performance) as well as improving match performances in the long term, also further analysis from current practices in (different) real world professional soccer settings - in which among other things, scientific considerations are not allowed to play the main/or any role at all - are of value. For example, they can either support the current state of knowledge, or conflicting findings can point to potential pitfalls in the successful implementation of, based on findings gained in more controlled settings, recommended methods in an ecological context or to deficiencies in the communication of scientific knowledge. Furthermore, when conducted regularly, investigations into the development of training methods over time become possible, also in relation to match performance.

Thus, the aim of this study is to quantify and compare the load that players experience in real world professional soccer training settings during drills that are i) frequently used, and ii) in which there is an intention to improve the players’ physical capacities (i.e., no drills focusing on, e.g., technical or tactical performance). For better contextualization, the days of a microcycle (i.e., periods lasting from the first to the last day focused on a match, and whose length may vary depending on the competitive calendar) on which these drills are performed are also to be evaluated.

## Materials and methods

### Participants

39 from a total of *N* = 43 elite male professional soccer field players, participating in training sessions from the first team of a Swiss club during the data collection period of the present study and for whom thereby training load data were recorded, were asked to take part in this study. Checks to ensure that there are no health contraindications for participation in training were carried out by the internal club sports medicine staff as part of their normal care of the team. Goalkeepers were excluded from the analysis due to their different activity profile compared to field positions (White et al., 2018), and four field players were not asked to participate since they left the club before the researchers had the opportunity to invite the eligible players. All players asked, or in the case of underage players, their legal guardians, voluntarily provided written informed consent. Although all data used in the current study arose from routine monitoring, which was part of the players’ professional employment (Winter and Maughan, 2009), ethical approval was obtained from a local ethics committee (Wrocław University of Health and Sport Sciences, Wrocław, Poland, identification number: 9/2023). After reducing the data set to the drills of interest (see section study design and research methods), the sample size considered for the analysis was *n* = 39.

### Study design and research methods

A retrospective observational cohort study was implemented over the course of 14 months, starting during the first half of the 2021/22 season and lasting until the winter break of the 2022/23 season. In this phase, the team under observation competed in the highest-level national championship (Credit Suisse Super League^®^) as well as the national cup competition (Helvetia Schweizer Cup) in Switzerland. In addition to 48 championship matches and 6 cup matches, there were 14 test matches.

The data analyzed derived from daily routine monitoring of the players. For the present study, drills were selected and summarized into categories as shown in Table 1. As it can be seen, in addition to a variety of sided games, high-intensity interval training (HIIT) drills were analyzed. Only data from on-field training drills performed in team sessions of the team under observation (i.e., rehabilitation sessions excluded) fitting into one of these categories were considered for analysis. It is worth noting that (probably) not all drills performed during the data collection period that could be assigned to one of these categories were included, but rather a random selection was given in the sense that only those drills whose characteristics could be verified (by L.A.) were included. Beyond this, data were considered regardless of whether, for example, a player was fielded at all or for how long in the previous or next match (Gualtieri et al., 2020), the microcycle structure (Anderson et al., 2016; Oliva-Lozano et al., 2022a) or situational and environmental conditions (Chmura et al., 2021; Oliveira et al., 2021; Oliva-Lozano et al., 2022b). The training sessions took place on natural grass pitches and artificial turf pitches, with the choice of pitch surface being made by the staff depending on weather conditions, infrastructural conditions, and the next match.

**TABLE 1:** Analyzed drill categories and their characteristics. In all forms of sided game, additional balls were distributed around the field to replace balls that went out—with the objective of maintaining intensity. Joker: neutral in-field player belonging to the team in ball possession; outside floater: neutral player slightly outside the field belonging to the team in ball possession. Thus, jokers and outside floaters imply a momentary numerical superiority of the team in ball possession. Formats always present the number of outfield players (i.e., excluding goal keepers).

Drill category	Objective, materials, field dimensions, rules	Typical duration and repetitions	Included observed formats
Goal-oriented large	With regular goals and goal keepers. Field dimensions approximately box to box. In some cases, the corners of the rectangular field were cut off, i.e., the field had the shape of a still strongly rectangular octagon. Offside rule present. Situationally regular restrictions such as limited number of ball touches or conditions for successful goal scoring.	2 to 4 games of 3 to 6 min	8vs.8, 8vs.8 plus 1 joker, 9vs.9, 9vs.9 plus 1 joker, 10vs.10, 10vs.10 plus 1 joker, 10vs.10 plus 2 joker
Goal-oriented medium	With regular goals and goal keepers. Field dimensions approximately half pitch. Situationally regular restrictions such as limited number of ball touches or conditions for successful goal scoring.	2 to 4 games of 2.5 to 6 min	6vs.5, 7vs.7, 8vs.8, 8vs.8 plus 1 joker, 8vs.8 plus 2 joker, 9vs.9 plus 1 joker, 9vs.9 plus 2 joker, 10vs.10, 10vs.10 plus 1 joker
Goal-oriented small no outside floaters	With regular goals and goal keepers but with more reduced field dimensions compared to large and medium goal-oriented games.	3 to 6 games of 1.5 to 6 min	3vs.3, 4vs.4, 5vs.5, 5vs.5 plus 1 joker, 6vs.6, 6vs.6 plus 2 joker
Goal-oriented small with outside floaters	With regular goals and goal keepers but with more reduced field dimensions compared to large and medium goal-oriented games. With outside floaters.	3 to 5 games of 0.75 to 4.25 min	3vs.3, 4vs.4, 5vs.5, 6vs.6; with number of supports ranging from 3 to 12
Goal-oriented mini-goals	With two or three mini-goals on two or four sides of a rectangular field and without goal keepers. Situationally regular restrictions such as limited number of ball touches or conditions for successful goal scoring.	2 to 4 games of 2.5 to 7.5 min	8vs.8 plus 1 joker, 8vs.8 plus 2 joker, 9vs.9, 9vs.9 plus 2 joker, 9vs.9 plus 3 joker, 10vs.10 plus 2 joker
Possession-oriented large	The aim is to maintain possession of the ball for as long as possible and “score” by either completing a given number of passes or passing the ball flat between two marks (multiple options). The opposition team is instructed to win the ball and instantly switch focus to maintain possession and “score”.	2 to 6 games of 2.25 to 4.5 min	8vs.8 plus 1 joker, 8vs.8 plus 2 joker, 8vs.8 plus 3 joker, 9vs.9, 10vs.10, 10vs.10 plus 1 joker, 10vs.10 plus 2 joker, 11vs.11
Possession-oriented medium	Same as possession large, but with more reduced field dimensions.	4 to 6 games of 1 to 3 min	4vs.4 plus 1 joker, 4vs.4 plus 2 joker, 4vs.4 plus 3 joker, 5vs.5 plus 1 joker, 5vs.5 plus 2 joker, 5vs.5 plus 3 joker, 6vs.6 plus 1 joker, 6vs.6 plus 2 joker, 6vs.6 plus 3 joker, 7vs.7 plus 2 joker, 7vs.7 plus 3 joker
HIIT	High-intensity intermittent interval training (HIIT). Short bouts of intended high-intensity running interspersed with passive recovery periods. Without ball.	1 or 2 series of 6 to 12 min	e.g., (combinations of) 10:20 s, 15:15 s, 5:25 s work:relief ratio

All drills were classified according to the number of days before or after a match day (i.e., match day (md) minus or plus) they were performed, with the assignment to a match chosen according to the focus of a training session. For example, md-2 means a training session focused on the upcoming match and took place 2 days before match day. In training sessions classified as match day plus one (md+1), the players with little playing time (situationally defined by the coaching staff) in the preceding match trained on the pitch, while the players with more playing time followed an individual regeneration program off the pitch. The label “general” was assigned if a training session was not focused on a match, this was the case, for example, in the first part of international breaks or in pre-season.

### External and internal load measures

A variety of external and internal load measures were monitored for each player using global navigation satellite system (GNSS) technology (Apex Pro, STATSports, Newry, Ireland) with 10 Hz sampling and heart rate chest straps (Polar H10, Polar Electro Oy, Kempele, Finland). In comparison with ECG, heart rate measurement derived from Polar H10 chest strap devices showed high correlation (*R* ^*2*^ = 1.00, *p* < .001) as well as minimal bias and limits of agreement during (cycling) exercise (Schaffarczyk et al., 2022). The validity and reliability of the STATSports Apex 10 Hz system were previously reported (Beato et al., 2018; Beato and de Keijzer, 2019; Crang et al., 2022). Apex 10 Hz is a multi GNSS augmented unit, capable of acquiring and tracking multiple satellite systems (e.g., GPS, GLONASS, Galileo, BeiDou) concurrently to provide the best possible position information. The Apex GNSS model reports information about the number of satellites connected (*M* = 13.9, *SD* = 1.8, range 9–22), which was lower than reported in previous literature (Beato et al., 2018; Beato and de Keijzer, 2019; Ammann and Altmann, 2023; Ammann et al., 2023b).

The Apex units present the following characteristics: 30 mm (wide) × 80 mm (high) dimensions, 48 g weight, 100 Hz gyroscope, 100 Hz tri-axial accelerometer, and 10 Hz magnetometer. For each player, an Apex unit was placed, according to manufacturer’s instructions, on the upper back between the right and left scapula through a manufacturer-provided vest. Underneath the vest, a heart rate chest strap, with the sensor measuring 65 mm (wide) × 34 mm (high) × 10 mm (thickness) and weighing 60 g, was worn. During data collection on the pitch, the heart rate data were also temporarily stored on the Apex unit. To avoid inter-unit errors, players used the same Apex units, vests, and heart rate chest straps for each session (Bourdon et al., 2017). After data collection on the pitch, the Sonra software (Sonra 4.0, STATSports, Newry, Ireland) was used to download all data recorded by the Apex unit and precisely define the drills of each player. The data was then exported as a csv file for further analysis.

The 9 load measures listed in Table 2 were selected for analysis, because they have been used frequently in practice and in studies analyzing load (especially in soccer), and literature proposes to consider them (Rago et al., 2019; Rago et al., 2020; Miguel et al., 2021; Teixeira et al., 2021). All load measures were expressed as frequency per minute to improve comparability between drills of different durations. The time ≥90% of maximal heart rate (HR_max_) is of interest as it is seen as an approximation for the time ≥90% of maximal oxygen uptake. The latter is considered as a large stimulus to elicit cardiovascular and peripheral adaptations leading to a higher maximal oxygen uptake (VO_2max_) capacity, a main factor of (endurance) exercise performance (Joyner and Coyle, 2008; Laursen, 2010; Buchheit and Laursen, 2013). The percentage thresholds applied for the relative speed thresholds (i.e., 55 and 70) are explained by the fact that they correspond to the recommended fixed thresholds (Miguel et al., 2021) for a maximum speed of 36 km/h. In the present analysis, the individual maximum speed was defined as the respective highest speed measured by GNSS (Massard et al., 2017), provided it followed a proper acceleration phase, the absence of which reveals clear measurement errors. In case a new maximum speed was measured, the new value replaced the previous one.

**TABLE 2:** Definitions and units of the external and internal load measures included in the analysis. a.u. = arbitrary units; HRmax = individual maximal heart rate.

Load measure	Unit	Definition
External load measures		
Accelerations	[n]	Acceleration efforts performed between 3 and 10 m/s^2^ with a minimum duration of 0.5 s
Decelerations	[n]	Deceleration efforts performed between 3 and 10 m/s^2^ with a minimum duration of 0.5 s
High-speed distance		
absolute	[m]	Distance ≥19.8 km/h (5.5 m/s)
relative	[m]	Distance ≥55% of individual maximal speed
Sprint distance		
absolute	[m]	Distance ≥25.2 km/h (7 m/s)
relative	[m]	Distance ≥70% of individual maximal speed
Total distance	[m]	Total distance covered
Internal load measures		
Edwards training load	[a.u.]	The sum of the activity time, in minutes, in each of the following five heart rate zones, multiplied by a factor to each zone: 50%–60% HR_max_ factor 1; 60%–70% HR_max_ factor 2; 70%–80% HR_max_ factor 3; 80%–90% HR_max_ factor 4; 90%–100% HR_max_ factor 5
Time ≥90% HR_max_	[min]	Time spent ≥90% of HR_max_

### Statistical analyses

All data were analyzed with the open-source software RStudio (R version 4.2.3 (2023-03-15 ucrt), Posit team 2023, Boston, United States). The descriptive statistics mean (SD) and range were used to describe and characterize the participants, while the descriptive statistics frequency and percentage were used to report the days of a microcycle on which certain drills were performed.

The assumptions of parametric one-way ANOVA tests were checked for each load measure to determine whether these tests can be used to assess whether statistically significant differences in load exist between drill categories. Based on Q-Q plots and Shapiro-Wilk tests, the assumption of normal distribution of the residuals was judged not to hold for all load measures. Based on Tukey-Anscombe plots, Levene’s tests and F_max_ tests, the assumption of homogeneity of variance of the residuals was judged to be severely violated for all load measures. Thus, for each load measure, a non-parametric Kruskal–Wallis test was calculated to assess whether statistically significant differences in load exist between drill categories. Eta squared (η^2^) based on the H-statistic with 95% confidence interval was computed as an effect size for Kruskal–Wallis rank sum tests (Tomczak and Tomczak, 2014). Two-sided Wilcoxon rank-sum tests with Bonferroni corrections for multiple testing were computed to evaluate between group differences. The correlation coefficient *r* with 95% confidence interval was computed as an effect size for the Wilcoxon rank sum tests as Z statistic divided by square root of the sample size (Tomczak and Tomczak, 2014). The 95% CI for eta squared (η^2^) and *r* were estimated using a bootstrap method (bootstrap percentile method with 1′000 random bootstrap samples). The level of significance was set at *p* < 0.05 for all tests.

For each load measure assessed, the load by drill category was visually represented using several descriptive methods. On the one hand, each observed drill was represented by a dot; additionally, the distribution of all corresponding drills was compactly visualized with boxplots showing the summary statistics median, first and third quartiles (i.e., Q1 the 25th percentile and Q3 the 75th percentile), and whiskers extending until the largest value no further than 1.5 * inter-quartile range (IQR; i.e., Q1 to Q3) from the hinge; and the distribution of all corresponding drills was further indicated by a probability density function.

## Results

Taken the data from each participant’s last drill in the data set, the average height of the 39 professional soccer players was = 1.808 m (*SD* = 0.072 m, range = 1.66 to 1.96 m), the average body weight was = 75.83 kg (*SD* = 7.01 kg, range = 63.4 to 89.4 kg). The mean age of the participants was = 23.63 years (*SD* = 4.56 years, range = 17.5 to 37.2 years) and an average individual maximal speed of = 34.252 km/h (*SD* = 1.537 km/h, range = 31.60 to 37.40 km/h) was recorded. Frequency and percentage of observed drills per day of a microcycle are shown in Table 3 for each drill category. Data in the drill categories goal-oriented mini-goals and goal-oriented small no outside floaters originated from 37 distinct players; 38 distinct players were observed for goal-oriented large, goal-oriented small with outside floaters, HIIT, possession-oriented large; and 39 players for goal-oriented medium as well as possession-oriented medium, respectively.

**TABLE 3:** Frequency and percentage (presented as n (%)) of observed drills per drill category and day of a microcycle. Md: match day.

Day in micro-cylce	Goal-oriented large (*n* = 1,576)	Goal-oriented medium (*n* = 910)	Goal-oriented small no outside floaters (*n* = 1,326)	Goal-oriented small with outside floaters (*n* = 2,389)	Goal-oriented mini-goals (*n* = 489)	Possession-oriented large (*n* = 1,661)	Possession-oriented medium (*n* = 2,607)	HIIT (*n* = 700)
general	480 (30.5%)	250 (27.5%)	285 (21.5%)	561 (23.5%)	178 (36.4%)	469 (28.2%)	550 (21.1%)	172 (24.6%)
md-5	0 (0%)	40 (4.4%)	0 (0%)	0 (0%)	60 (12.3%)	163 (9.8%)	0 (0%)	71 (10.1%)
md-4	616 (39.1%)	80 (8.8%)	48 (3.6%)	301 (12.6%)	251 (51.3%)	685 (41.2%)	964 (37.0%)	306 (43.7%)
md-3	440 (27.9%)	521 (57.3%)	290 (21.9%)	100 (4.2%)	0 (0%)	306 (18.4%)	424 (16.3%)	21 (3.0%)
md-2	40 (2.5%)	0 (0%)	0 (0%)	0 (0%)	0 (0%)	0 (0%)	164 (6.3%)	15 (2.1%)
md-1	0 (0%)	19 (2.1%)	0 (0%)	1,305 (54.6%)	0 (0%)	38 (2.3%)	0 (0%)	25 (3.6%)
md+1	0 (0%)	0 (0%)	703 (53%)	122 (5.1%)	0 (0%)	0 (0%)	505 (19.4%)	90 (12.9%)

As shown in Table 4, the load experienced by players during the drills was statistically significant different between the drill categories for each assessed load measure. Two-sided Wilcoxon rank-sum tests with Bonferroni corrections revealed statistically significant differences in load between most drill categories for all load measures assessed. The estimated median of the difference between a sample from drill category x and a sample from drill category y, the W test statistic, the adjusted *p*-value, and the effect size *r* with 95% CI are reported in the Supplementary Materials.

**TABLE 4:** Kruskal–Wallis tests between drill categories for each load measure assessed.

Load measure	*n*	Statistic	df	*p*	η^2^ [95% CI]
External load measures					
Accelerations	11,658	2035.7	7	<.001*	0.17 [0.16 to 0.19]
Decelerations	11,658	3001.9	7	<.001*	0.26 [0.24 to 0.27]
High-speed distance					
absolute	11,658	4616.4	7	<.001*	0.40 [0.38 to 0.41]
relative	11,658	4604.3	7	<.001*	0.39 [0.38 to 0.41]
Sprint distance					
absolute	11,658	2043.6	7	<.001*	0.17 [0.16 to 0.19]
relative	11,658	3300.7	7	<.001*	0.28 [0.27 to 0.30]
Total distance	11,658	3228.9	7	<.001*	0.28 [0.26 to 0.29]
Internal load measures					
Edwards training load	11,658	1,251.2	7	<.001*	0.11 [0.10 to 0.12]
Time ≥90% HR_max_	11,658	879.8	7	<.001*	0.07 [0.07 to 0.09]

Confidence level of the eta squared (η2) confidence intervals = 0.95. Level of significance: *p < 0.05.

**FIGURE 1:**
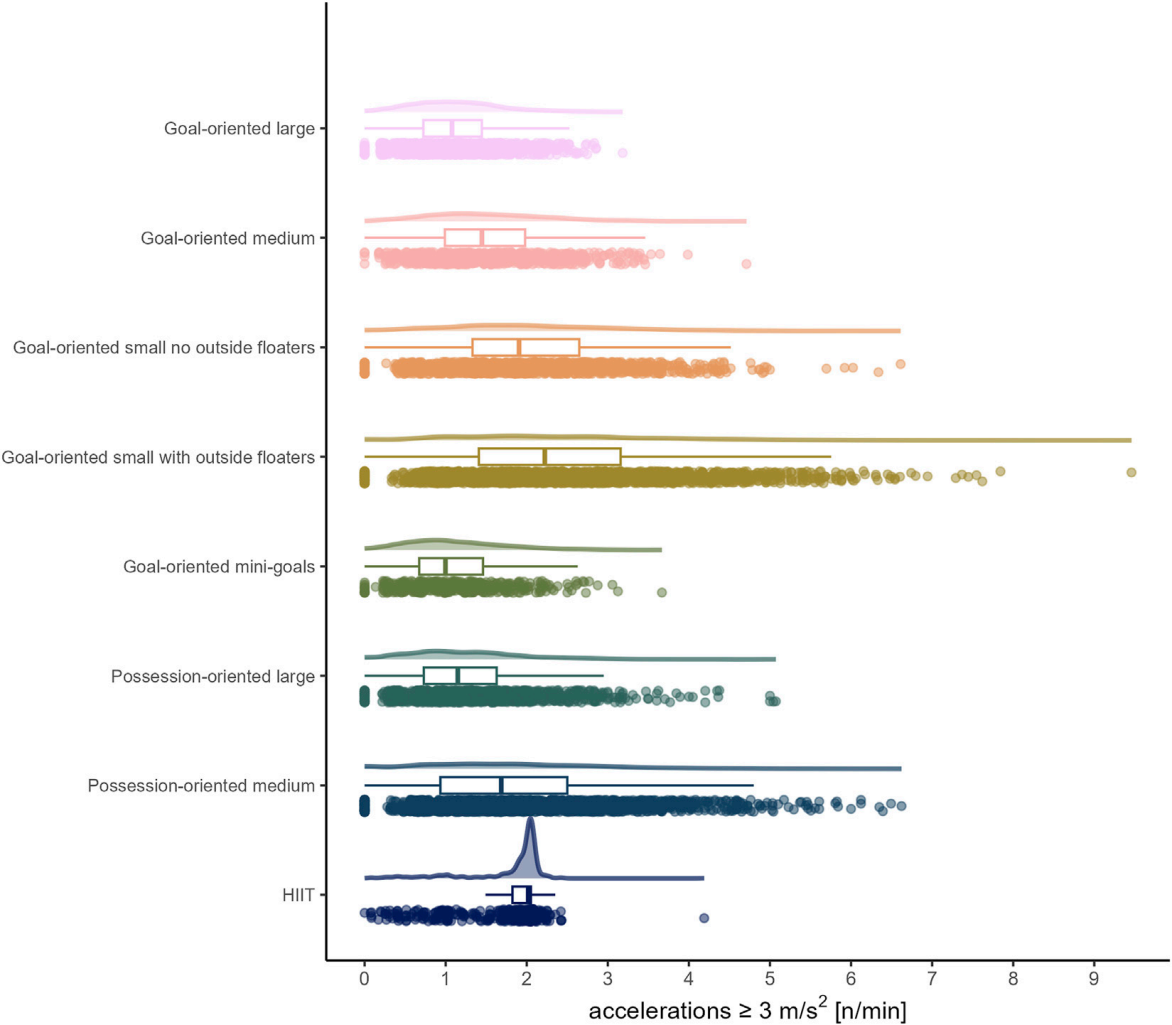
Accelerations per minute performed by players during the observed drills, presented by drill category. Each dot represents an observed drill; boxplots, showing the median, first and third quartiles, and whiskers extending until the largest value no further than 1.5 * inter-quartile range from the hinge, provide information on the distribution of all corresponding drills; which is further indicated by a probability density function.

**FIGURE 2:**
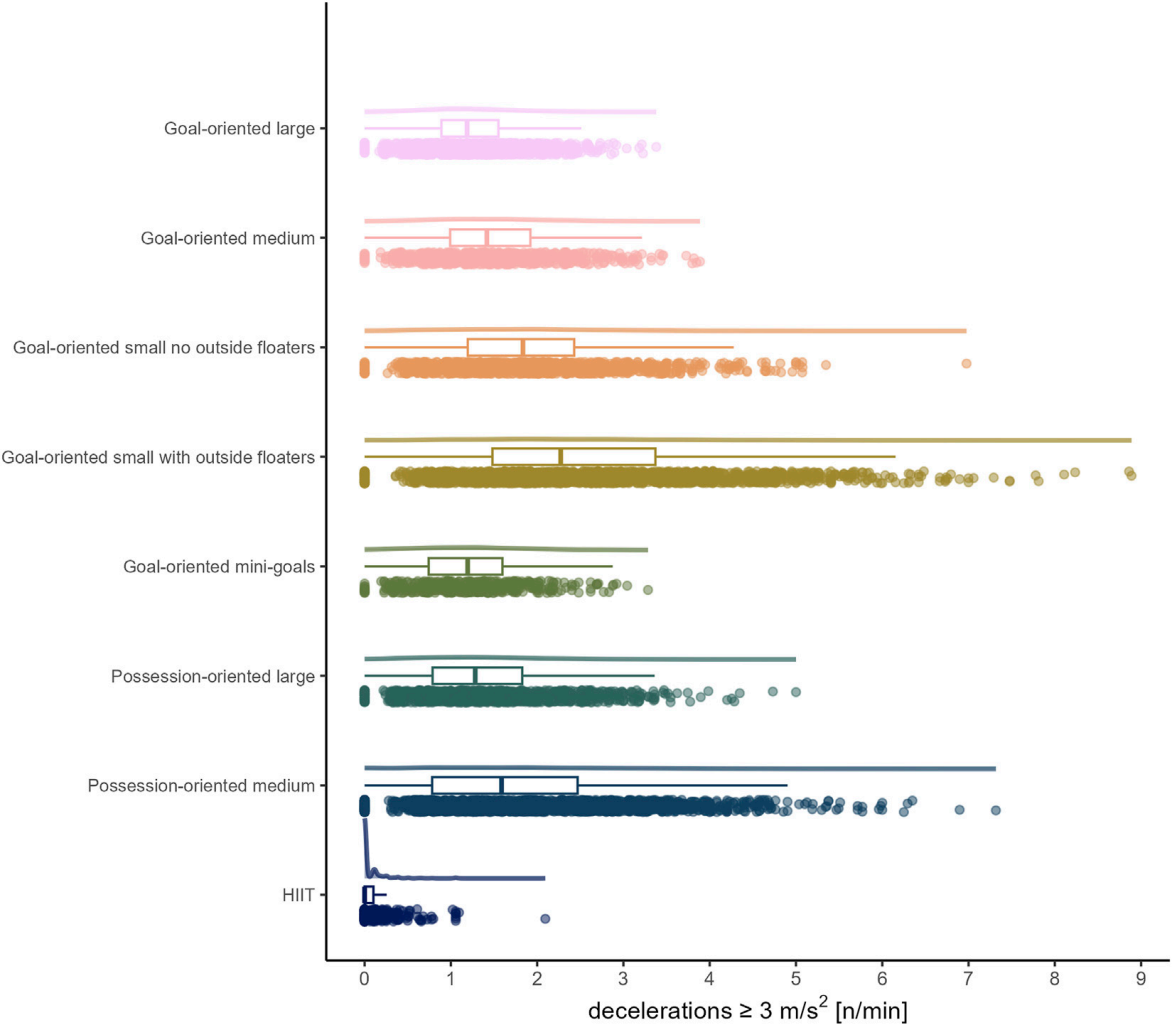
Decelerations per minute performed by players during the observed drills, presented by drill category. Each dot represents an observed drill; boxplots, showing the median, first and third quartiles, and whiskers extending until the largest value no further than 1.5 * inter-quartile range from the hinge, provide information on the distribution of all corresponding drills; which is further indicated by a probability density function.

**FIGURE 3:**
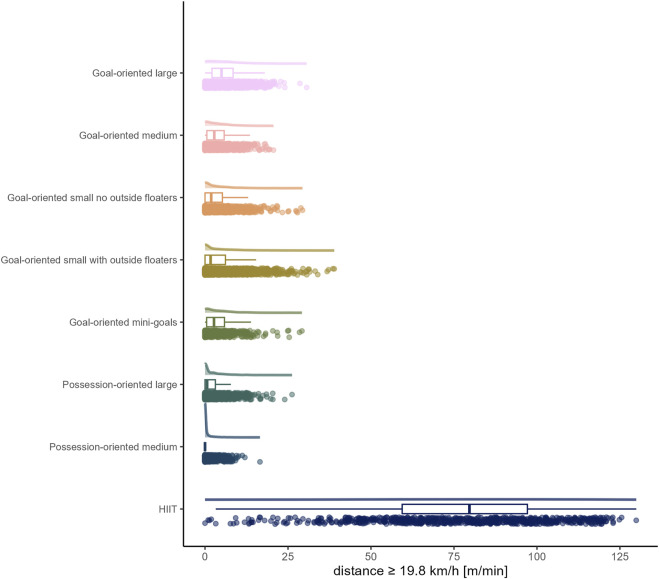
Absolute high-speed distance [m] per minute performed by players during the observed drills, presented by drill category. Each dot represents an observed drill; boxplots, showing the median, first and third quartiles, and whiskers extending until the largest value no further than 1.5 * inter-quartile range from the hinge, provide information on the distribution of all corresponding drills; which is further indicated by a probability density function.

**FIGURE 4:**
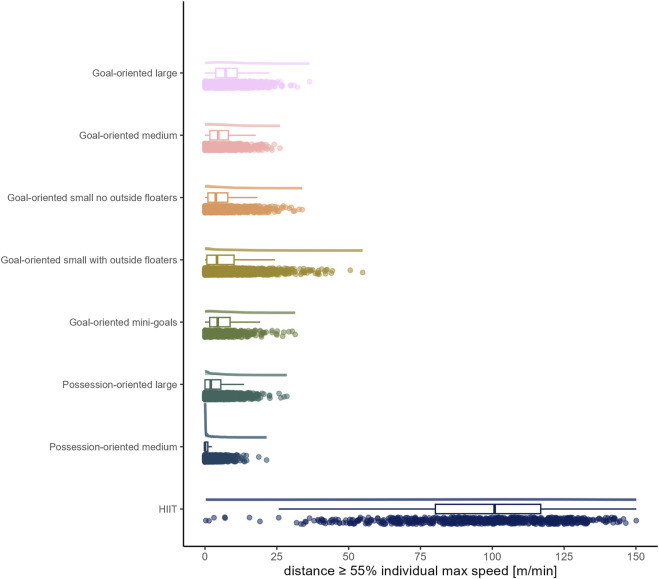
Relative high-speed distance [m] per minute performed by players during the observed drills, presented by drill category. Each dot represents an observed drill; boxplots, showing the median, first and third quartiles, and whiskers extending until the largest value no further than 1.5 * inter-quartile range from the hinge, provide information on the distribution of all corresponding drills; which is further indicated by a probability density function.

**FIGURE 5:**
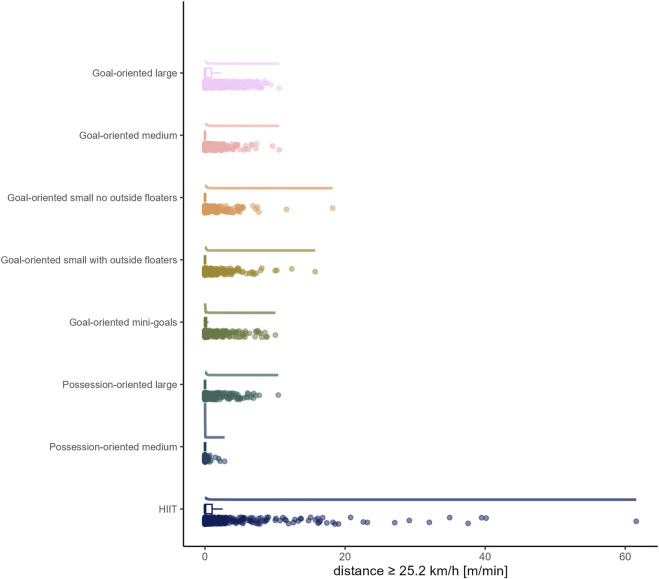
Absolute sprint distance [m] per minute performed by players during the observed drills, presented by drill category. Each dot represents an observed drill; boxplots, showing the median, first and third quartiles, and whiskers extending until the largest value no further than 1.5 * inter-quartile range from the hinge, provide information on the distribution of all corresponding drills; which is further indicated by a probability density function.

**FIGURE 6:**
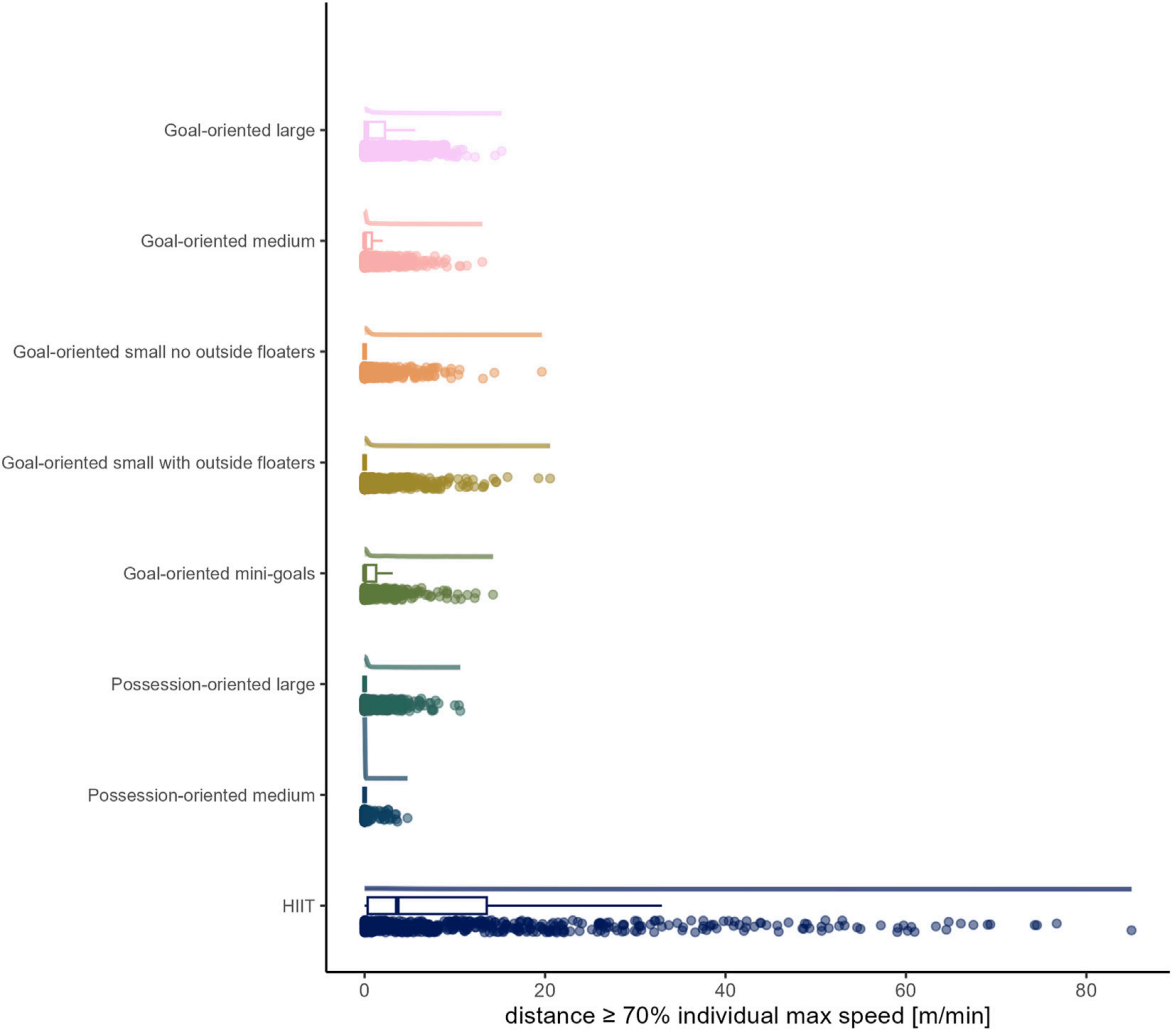
Relative sprint distance [m] per minute performed by players during the observed drills, presented by drill category. Each dot represents an observed drill; boxplots, showing the median, first and third quartiles, and whiskers extending until the largest value no further than 1.5 * inter-quartile range from the hinge, provide information on the distribution of all corresponding drills; which is further indicated by a probability density function.

**FIGURE 7:**
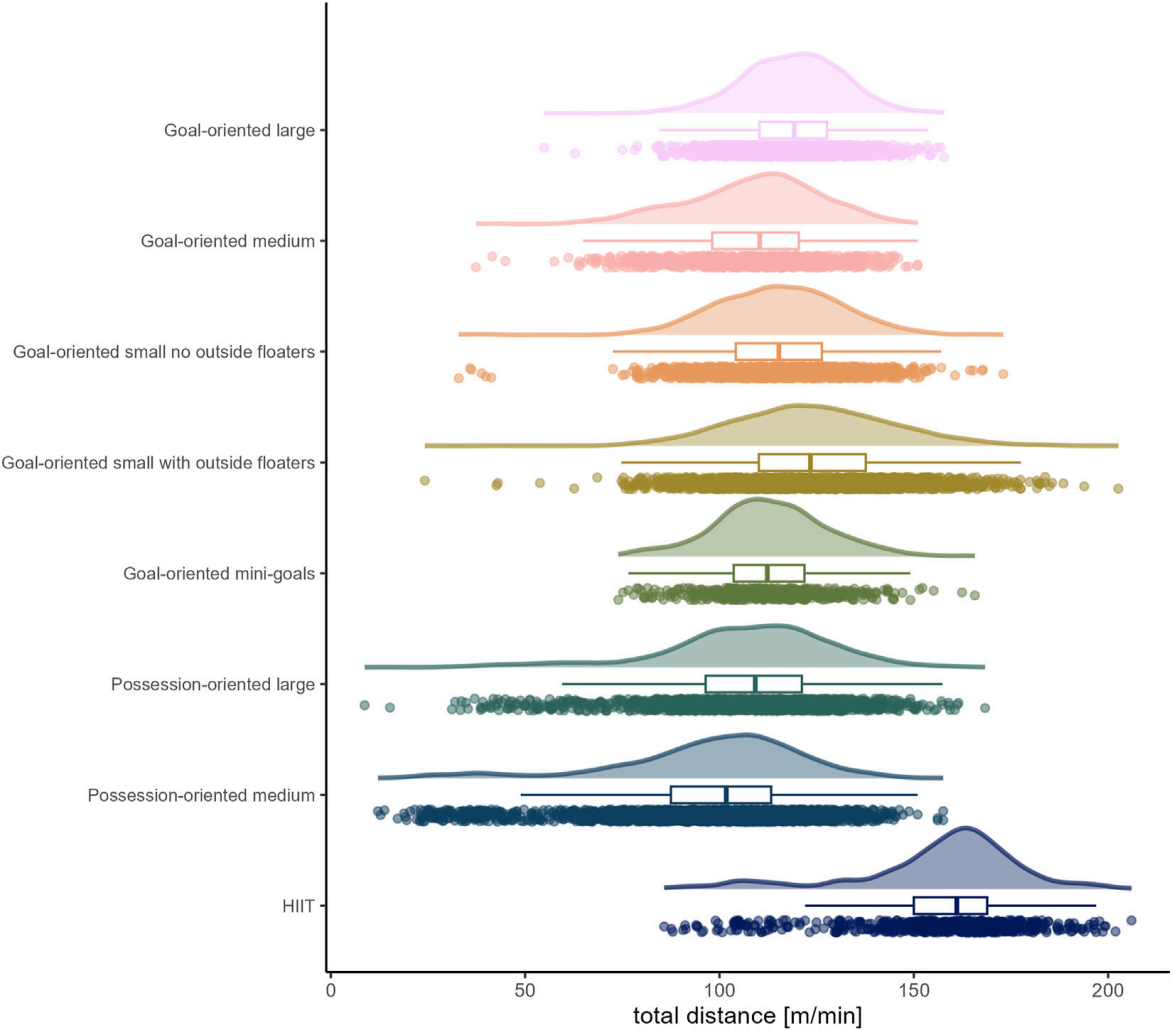
Total distance [m] per minute performed by players during the observed drills, presented by drill category. Each dot represents an observed drill; boxplots, showing the median, first and third quartiles, and whiskers extending until the largest value no further than 1.5 * inter-quartile range from the hinge, provide information on the distribution of all corresponding drills; which is further indicated by a probability density function.

**FIGURE 8:**
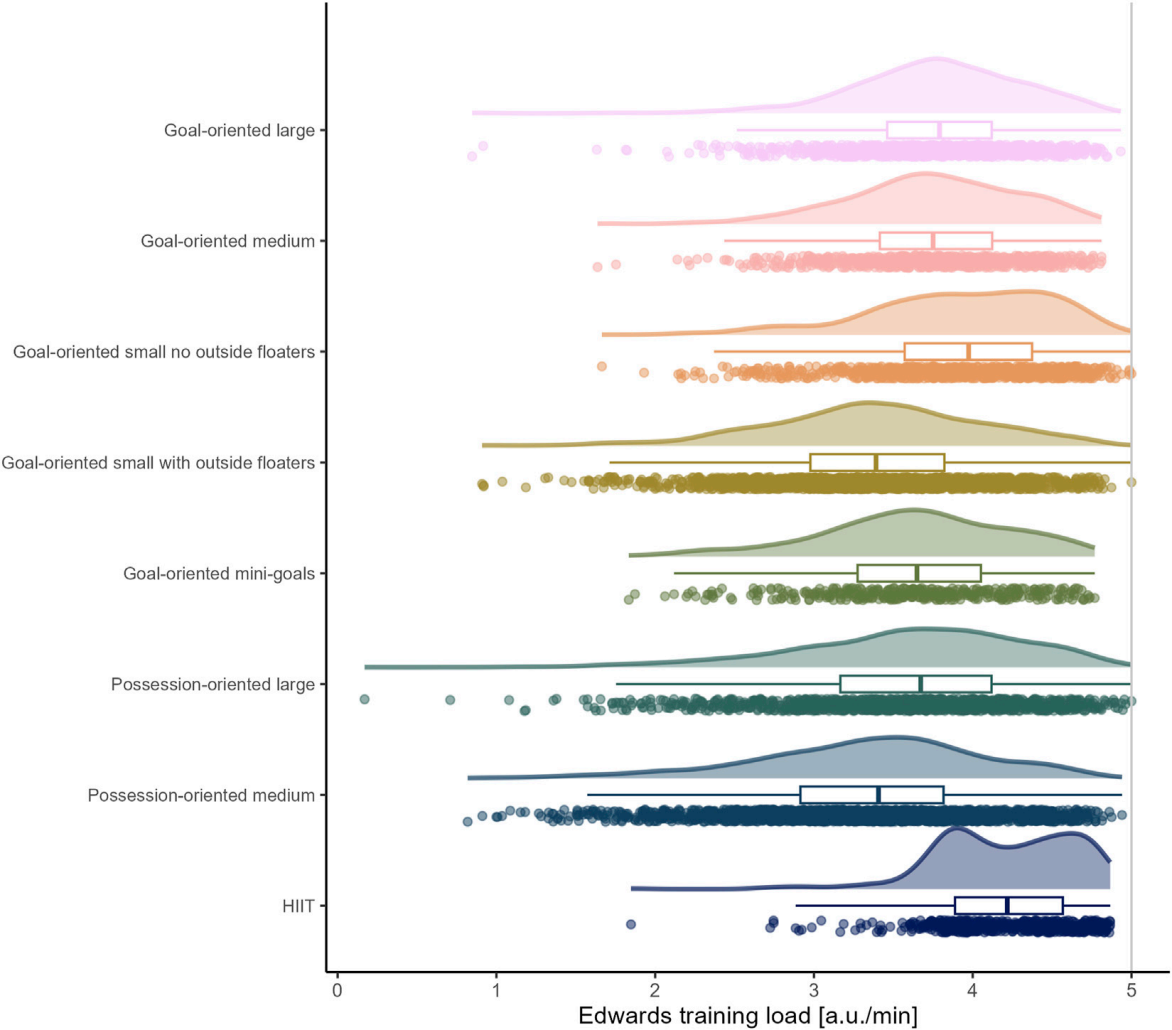
Edwards training load per minute experienced by players during the observed drills, presented by drill category. Each dot represents an observed drill; boxplots, showing the median, first and third quartiles, and whiskers extending until the largest value no further than 1.5 * inter-quartile range from the hinge, provide information on the distribution of all corresponding drills; which is further indicated by a probability density function.

**FIGURE 9:**
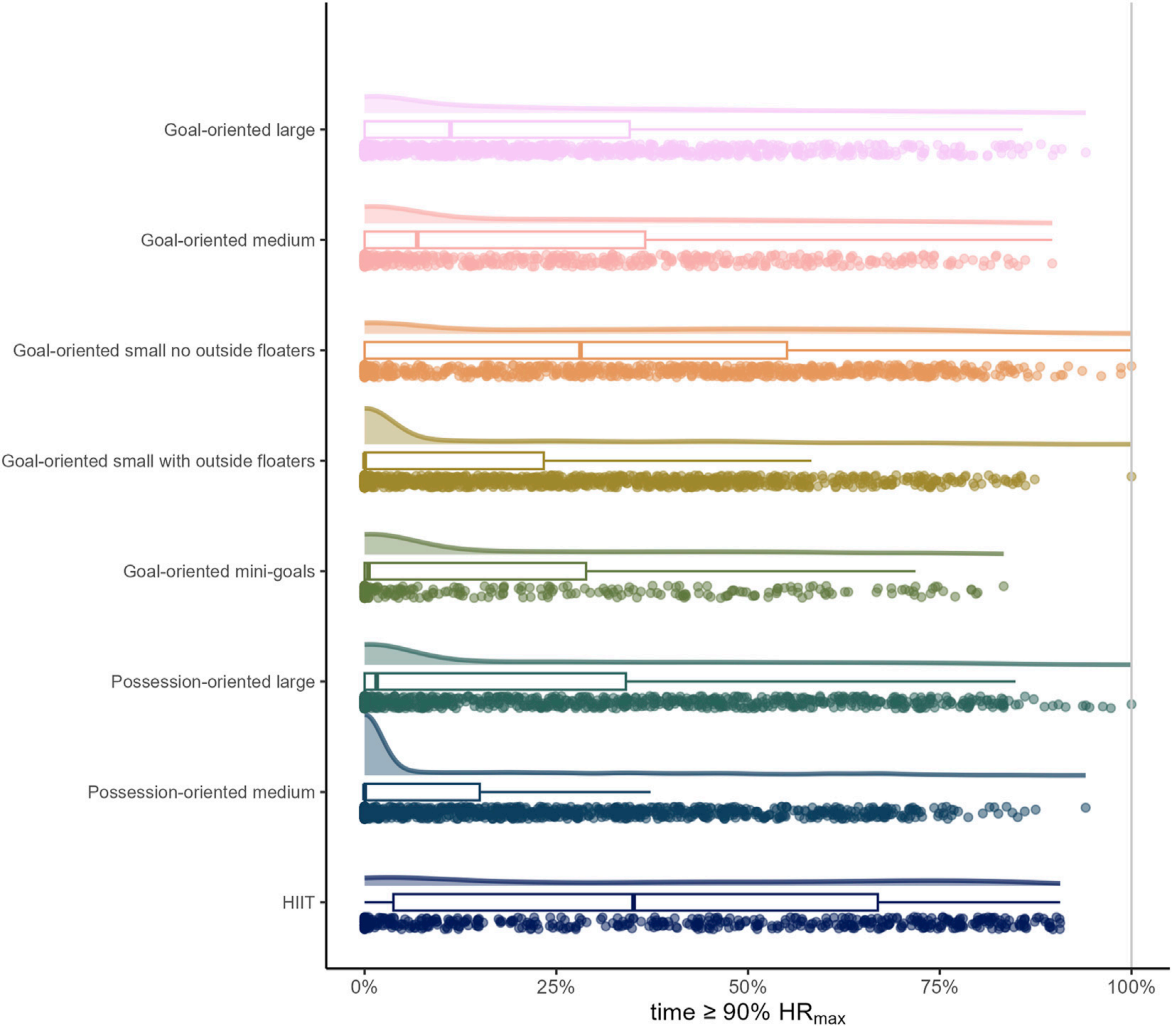
Percentage of time that players spent at an intensity ≥ 90% HR_max_ during the observed drills, presented by drill category. Each dot represents an observed drill; boxplots, showing the median, first and third quartiles, and whiskers extending until the largest value no further than 1.5 * inter-quartile range from the hinge, provide information on the distribution of all corresponding drills; which is further indicated by a probability density function.

The load by drill category is visually represented in Figure 1 for accelerations, Figure 2 for decelerations, Figure 3 for absolute high-speed distance, Figure 4 for relative high-speed distance, Figure 5 for absolute sprint distance, Figure 6 for relative sprint distance, Figure 7 for total distance, Figure 8 for Edwards training load, and Figure 9 for the time ≥90% HR_max_.

## Discussion

The aim of this study was to quantify and compare the load that players experience in real world professional soccer training settings during drills that are i) frequently used, and ii) in which there is an intention to improve the players’ physical capacities. For better contextualization, the days of a microcycle on which these drills are performed were also to be evaluated.

### Total distance

The players in the present observation covered considerably more distance per minute in HIIT drills (*median* (*IQR*) = 161.06 m (150.00 to 168.88 m)) than in all sided games. When focusing on sided games, existing literature indicates the distance covered per minute by players is reduced as the relative pitch area (i.e., area per player) gets smaller (Beato et al., 2023; Clemente et al., 2023) and that the distance covered per minute can be affected by the rules of the game (Clemente et al., 2021; Beato et al., 2023). In the present study, it was not possible to calculate the relative pitch area in each case; the size indicators in the drill category names refer to both the number of players and the field dimensions. However, regarding these size aspects, players covered less distance per minute in the medium forms of both game-oriented (*median* (*IQR*) = 110.35 m (98.13 to 120.40 m)) and possession-oriented (*median* (*IQR*) = 101.74 m (87.50 to 113.30 m)) sided games than in their larger counterparts (*median* (*IQR*) = 119.23 m (110.26 to 127.67 m), *W* = 963,853.5, *p* < .001, *r* = 0.29, 95% CI [0.25, 0.32]; and *median* (*IQR*) = 109.21 m (96.43 to 121.20 m), *W* = 2,670,560.0, *p* < .001, *r* = 0.20, 95% CI [0.17, 0.23]; respectively). Interestingly, in small goal-oriented games, players covered a greater distance when outside floaters were present compared to these games without outside floaters.

While the present analysis shows a larger number of drills with a lower distance per minute for possession-oriented sided games compared to the goal-oriented sided games, existing findings are not consistent as to whether, and if so, which of these two game orientations is associated with a higher distance covered per minute (Clemente et al., 2021; Beato et al., 2023). One possible explanation for the present observation could be that in possession-oriented games, in which scoring can be achieved by a certain number of successive passes, players without possession of the ball consider the risk of suffering a “goal” to be lower (due to the attacking team’s own mistakes) than in goal-oriented games and therefore behave more passively. Moreover, in possession-oriented games, players might more likely consider “hiding” (Hill-Haas et al., 2011).

### High-speed running and sprinting

In accordance with previous research (Beato et al., 2023; Dello Iacono et al., 2023), distances covered per minute at both absolute and relative high-speed proved to be limited in all sided games (range of medians for absolute high-speed distance: 0 to 4.97 m and for relative high-speed distance: 0 to 7.14 m). However, as the raw data show, and what is thus a valuable addition to the existing literature, there were still drills/players, with up to maximally about 50 m covered at high-speed per minute, only a small proportion of which also fell into the sprint domain. On the contrary, HIIT drills forced players to cover distance at high-speed (absolute high-speed: *median* (*IQR*) = 79.66 m (59.40 to 97.12 m); relative high-speed: *median* (*IQR*) = 100.81 m (80.19 to 116.86 m)), whereby for some players and/or drills sprinting was observed. The observation of nearly no sprinting distance in all sided games is in line with existing literature (Beato et al., 2023; Dello Iacono et al., 2023). The present data furthermore indicate both high-speed and sprint distances in sided games decrease with reduced size indicators of pitch area and number of players. Likewise, a meta-analytical comparison by Clemente et al. (2023) indicated an increase of high-speed running distance with a larger pitch size and meta-regressions conducted by Dello Iacono et al. (2023), similar to total distance per minute, suggest the relative pitch area as an important moderating variable for both high-speed and sprint distances.

### Accelerations and decelerations

Looking at Figures 1, 2, the number of accelerations and decelerations appear to be similar within each sided game, whereas this is not the case for HIIT, which in addition yielded hardly any deceleration. The latter finding is important to be aware of, as the game of soccer requires players to decelerate repeatedly (Harper et al., 2019; Silva et al., 2022; Ammann et al., 2023a; Ammann and Altmann, 2023) and the literature suggests that performing decelerations is a plausible way to prepare players for them (Harper et al., 2019; Silva et al., 2022). While existing literature (Silva et al., 2022) reports accelerations and decelerations to be higher in sided games compared to other methods such as circuit training or running-based drills, to which HIIT drills are often classified, this does not generally apply to the accelerations in the present data. In line with previous research (Silva et al., 2022) accelerations and decelerations were found to typically increase with decreasing format of the sided game (e.g., reduced number of players, smaller pitch size). While the summary statistics in the present observation are similar to those of previous research (e.g., Beato et al., 2023), the current data emphasize the relevance of individual load management in real world professional soccer settings by revealing a greater spread.

### Time ≥90% HR_max_ and Edwards training load

From the drills assessed in the present analysis, HIIT drills were those in which players typically spent the greatest percentage of time at an intensity ≥90% HR_max_. However, the raw data also reveal numerous drills/players in the sided games with an enhanced percentage of time at an intensity ≥90% HR_max_ as well as drills/players in the HIIT category with only a low percentage of time at an intensity ≥90% HR_max_. In general, a wide range of data can be observed in each drill category. Given the heart rate lag at exercise onset, it is imperative to consider exposure-related aspects (e.g., duration of drill, between-drills duration, number of consecutive drills) of the analyzed drills when interpreting the present data (Buchheit and Laursen, 2013). While the HIIT drills typically lasted between six to 12 minutes and no more than two drills followed each other, the sided games typically had comparatively shorter durations and were repeated more frequently. The Edward training load data in Figure 8 show that the exposure-related aspects were defined in such a way that elevated heart rates could be observed for most drills/players. However, if an aim of the coaches was to increase the players’ maximal oxygen update (VO_2max_) capacity with the assessed drills, it can be criticized that better defined exposure-related aspects of the sided games and a greater individualization (e.g., fitness level, oxygen consumption kinetics) of the HIIT drills might have been more effective for this purpose.

Regarding the drills classified as goal-oriented small with outside floaters, it might be important to consider that they were predominantly performed on md-1, as it seems likely the coaches were more concerned getting the players (mentally) “ready” with short, intense sequences than setting an effective physiological training stimulus.

It is both known that matches typically provide a strong cardio-vascular stimulus for players and that accelerations, decelerations, as well as distances covered–especially at high-speed and by sprinting - are key physical performance criterion for players in matches (Suarez-Arrones et al., 2015; Chmura et al., 2021; Forcher et al., 2022; Ammann et al., 2023a; Ammann and Altmann, 2023). Additionally, is reported to be common practice of coaches to implement so-called compensation training sessions, which aim to compensate for missed match load (Martin-Garcia et al., 2018; Casamichana et al., 2022; Ammann and Altmann, 2023; Ammann et al., 2023b). Based on this and findings of the present study, a combination of the two, cleverly modified, methods sided games and HIIT seems to be a sensible option for these sessions. Furthermore, while outside floaters were not examined in the present study, visual inspection suggests lower loads for this role. It seems essential for coaches to be aware of this and they may take advantage of it if appropriate, for example, by fielding rehabilitation players in these positions.

### Limitations, direction

In general, caution must be taken when generalizing the current findings, since data from only one male team were analyzed. Within this analysis it should be considered that (probably) not all drills performed during the data collection period that could be assigned to one of these categories were included as well as that data on internal load were not available for some drills. The latter was the case for inaccurate measurements of heart rate (visual assessment by LA). However, as these did not occur systematically, it was decided not to exclude drills with missing heart rate-based data in favor of a larger data set. Moreover, it would have been interesting to investigate additional measures of internal load (e.g., sRPE, blood lactate concentration) to get a more complete picture, and as heart rate may be influenced by factors such as emotions or caffeine (Hill-Haas et al., 2011; Miguel et al., 2021). However, the current real world professional soccer setting did not allow to measure them after every drill and routinely. Regarding accelerations and decelerations, it should be noted, while there is no doubt that these measures should be considered in professional load management (Harper et al., 2019; Delves et al., 2021; Miguel et al., 2021; Silva et al., 2022), it is less clear how to quantify such loads (Harper et al., 2019; Silva et al., 2022). As it is common in various studies and recommended in the literature, we employed threshold-based counts (Harper et al., 2019; Delves et al., 2021; Miguel et al., 2021). However, regarding accelerations, Sonderegger et al. (2019) showed that if the running speed immediately prior to an acceleration being initiated and the maximal acceleration capacity associated with it are not considered, a number of high-intensity accelerations could be missed. I.e., arbitrarily set thresholds lead to accelerations from low speeds being overestimated and accelerations from high speeds being underestimated.

## Conclusion

Based on our findings, we conclude that different drills provide different stimuli to players in real world professional soccer settings. More specifically, HIIT drills proved to be a more powerful tool of getting players to cover distances at high-speed (i.e., ≥19.8 km/h and ≥55% of individual maximal speed) and to spend time at an intensity ≥90% HR_max_ compared to sided games. However, our data reveal HIIT drills come with the drawback that players hardly perform any deceleration, and it is known that technical and tactical aspects cannot be addressed. The sprint distance of players was very low in all sided games and in most cases also in HIIT drills. In small goal-oriented sided games, players covered a greater distance per minute when outside floaters were present. Particularly regarding an improvement of the players’ aerobic capacity, the present data emphasize the relevance for coaches to ensure an appropriate exposure. In general, the importance of individual load management in professional soccer is highlighted.

## Data Availability

The datasets presented in this article are not readily available because in combination with free available data, the datasets allow identification of the participants. Requests to access the datasets should be directed to linda_ammann@gmx.ch.
